# Myoblast 3D bioprinting to burst in vitro skeletal muscle differentiation

**DOI:** 10.1002/term.3293

**Published:** 2022-03-04

**Authors:** Flavio L. Ronzoni, Flaminia Aliberti, Franca Scocozza, Laura Benedetti, Ferdinando Auricchio, Maurilio Sampaolesi, Gabriella Cusella, Itedale Namro Redwan, Gabriele Ceccarelli, Michele Conti

**Affiliations:** ^1^ Department of Public Health, Experimental and Forensic Medicine Human Anatomy Unit University of Pavia Pavia Italy; ^2^ Department of Biomedical Sciences Humanitas University Pieve Emanuele Italy; ^3^ Fondazione IRCCS Policlinico San Matteo Center for Inherited Cardiovascular Diseases Transplant Research Area Pavia Italy; ^4^ Department of Civil Engineering University of Pavia Pavia Italy; ^5^ Department of Development and Regeneration Translational Cardiomyology KU Leuven Leuven Belgium; ^6^ CELLINK AB Gothenburg Sweden

**Keywords:** commercially hydrogel bioinks, murine myoblasts (C2C12), muscle differentiation, three‐dimensional (3D) bioprinting

## Abstract

Skeletal muscle regeneration is one of the major areas of interest in sport medicine as well as trauma centers. Three‐dimensional (3D) bioprinting (BioP) is nowadays widely adopted to manufacture 3D constructs for regenerative medicine but a comparison between the available biomaterial‐based inks (bioinks) is missing. The present study aims to assess the impact of different hydrogels on the viability, proliferation, and differentiation of murine myoblasts (C2C12) encapsulated in 3D bioprinted constructs aided to muscle regeneration. We tested three different commercially available hydrogels bioinks based on: (1) gelatin methacrylate and alginate crosslinked by UV light; (2) gelatin methacrylate, xanthan gum, and alginate‐fibrinogen; (3) nanofibrillated cellulose (NFC)/alginate‐fibrinogen crosslinked with calcium chloride and thrombin. Constructs embedding the cells were manufactured by extrusion‐based BioP and C2C12 viability, proliferation, and differentiation were assessed after 24 h, 7, 14, 21, and 28 days in culture. Although viability, proliferation, and differentiation were observed in all the constructs, among the investigated bioinks, the best results were obtained by using NFC/alginate‐fibrinogen‐based hydrogel from 7 to 14 days in culture, when the embedded myoblasts started fusing, forming at day 21 and day 28 multinucleated myotubes within the 3D bioprinted structures. The results revealed an extensive myotube alignment all over the linear structure of the hydrogel, demonstrating cell maturation, and enhanced myogenesis. The bioprinting strategies that we describe here denote a strong and endorsed approach for the creation of in vitro artificial muscle to improve skeletal muscle tissue engineering for future therapeutic applications.

## 1 INTRODUCTION

Skeletal muscle has the great capacity to self‐repair and regenerate in response to common acute injuries, such as exercise‐induced damage (Giarratana et al., 2020; Ronzoni et al., 2021). This is principally due to a resident stem cell population that is mainly involved in skeletal muscle homeostasis and regeneration. It has been demonstrated that these muscular progenitor cells are able to fuse, forming myotubes even if treated with recombinant proteins (Agosti et al., 2020; Perini et al., 2015; Ronzoni et al., 2011, 2017). However, when muscle loss becomes irreversible (e.g., in case of severe trauma, invasive surgeries, degenerative diseases, or because of aging), lesions are so critical that they impair muscle functionality (Young, 1964). In this scenario, muscle regenerative medicine can provide solutions (Langridge et al., 2021; Ronzoni et al., 2020).

Several studies focused on the production of an ideal structure to induce muscle tissue regeneration, including biochemical components to ensure efficient myogenic differentiation and maturation, resulting in thick and elongated myotube formation (Kang et al., 2016). However, the current challenge is to ensure the uniform growth of muscle cells inside the biomaterial and to induce a contractile syncytium similar to the native skeletal muscle structure (Chen, 1993) despite, over the years, different biomaterials and scaffold designs have been experimentally and/or clinically evaluated for the repair of skeletal muscle tissue.

In particular, porous three‐dimensional (3D) scaffolds have been manufactured using natural or synthetic polymers (Melchels et al., 2012), hydrogels (Baar et al., 2005; Fedorovich et al., 2008; L’Heureux et al., 2006; Stevens et al., 2009; Visser et al., 2013), decellularized extracellular matrix (dECM), and their composites (Ott et al., 2008). Several advantages emerge from the use of such natural hydrogels, such as mimicking skeletal muscle environment, providing bioactive signaling for muscle differentiation, and reabsorbing the biomaterial to allow the in vivo interaction of myofibers (Lev & Seliktar, 2018). Advantageous is also the use of dECMs that preserve the native tissue architecture, facilitate the adhesion of muscular cells and promote the regeneration of the tissue area in which the damage is (Lev & Seliktar, 2018; Wolfe & Sell, 2011). Nevertheless, there are some limitations associated with the use of such natural materials; for instance, the inadequate supply of nutrients to the cells in the central portion of the bioconstruct or, regarding dECMs, long incubation times to observe the effective functional recovery of the damaged tissue is required (Smoak & Mikos, 2020). As for the synthetic polymeric matrices, they do not guarantee good cell adhesion, they are poorly absorbable and there is a greater risk of activation of immune response of the patients. Therefore they are not considered biocompatible (Lev & Seliktar, 2018).

Costantini et al. (2017) encapsulated C2C12 murine myoblast into gelatin methacryloyl hydrogel (CELLINK^®^ GelMA ‐ CELLINK AB, Gothenburg, Sweden) using 3D mold to evaluate 3D cell culture in terms of in vitro myogenesis; moreover, they demonstrated that both hydrogel stiffness and geometrical confinement play a crucial role in the differentiation of myogenic precursors in a three‐dimensional environment. Otherwise, Seyedmahmoud et al. (2019) encapsulated C2C12 not only in CELLINK^®^ GelMA, but also in CELLINK^®^ GelMA mixed with different percentages of alginate (6% and 8%). They demonstrated that alginate percentage can provide a more favorable mechanical microenvironment for murine myoblasts (C2C12) cell proliferation and an optimal niche to induce muscle tissue formation.

Bauer and colleagues (Costantini et al., 2018) demonstrated that spreading and proliferation of C2C12 cells encapsulated into alginate‐based hydrogel were impacted by both stiffness and stress relaxation behavior of the substrates created by 3D molding. In addition, Matthias et al. (Costantini et al., 2018) evaluated the efficacy of muscle‐derived stem cells combined with fibrin hydrogel for volumetric muscle loss repair using 3D mold. Among different materials, Garcia and colleagues used hyaluronic acid‐based hydrogel to realize scaffolds by 3D molding for mimicking the regenerative environment. Then, they seeded cells on them, to determine how the biomechanical properties differentially influence MP and connective tissue cells (Juan Martin Silva Garcia & Panitch, 2017).

Furthermore, thanks to the advancement of additive manufacturing, three‐dimensional bioprinting is nowadays a widely adopted technique for both manufacturing 3D scaffolds and constructs in various tissue engineering approaches (Nikolova & Chavali, 2019). In fact, BioP not only allows the production of scaffolds whose geometry can be controlled thanks to the use of specific software, but it can also be exploited for the manufacturing of different scaffolds based on different biomaterials in which different cell types can be encapsulated (Derby, 2012; Leong et al., 2003; Murphy & Atala, 2014). The outcome of BioP, which is a complex process defined by several steps, is conditioned by the printing technology and biomaterial adopted, which defines when combined with cells the so‐called bioink (Groll et al., 2019; Matai et al., 2020; Ng et al., 2019).

Bioprinting techniques can be classified according to the printing methods, in particular, it is possible to distinguish three main BioP techniques: inkjet, extrusion, and vat‐polymerization (AmerDababneh & Bioprinting Technology, 2014). These techniques vary in precision and accuracy in the deposition of the material, stability, and cell survival.

The inkjet‐based BioP was the first technique to be implemented. The bioink solution is manipulated by generating droplets which are deposited on a substrate using a small nozzle. The jet delivered can be of three types: continuous, on command (drop‐on‐demand) and electrodynamic (Gudapati et al., 2016). This technique offers many advantages thanks to its simplicity, versatility, and control in the bioink deposition of the allowing to control the bioink volume to be deposited. The disadvantage is that inkjet technique does not allow to process high viscosity bioink.

Extrusion‐based BioP is a combination of a pneumatic or mechanical fluid dispensing system and an automatic robotic system for the extrusion and the 3D printing (Jiang et al., 2019). The bioink is dispensed by a deposit system on a substrate on which, thanks to a light, chemical solutions or thermal transistors, the crosslinking of the bioink takes place, thus obtaining the deposition of cells encapsulated in cylindrical filaments, allowing the creation of 3D structures. The mechanical extrusion of the bioink solution involves the use of a piston or a screw, while the pneumatic extrusion involves the use of compressed air. Although the extrusion BioP is the most used technique in this field, there are some limitations for the realization of the desired structure such as the shear effort and the limited selection of the material due to the need to encapsulate the cells inside the bioink and its rapid gelling.

Vat polymerization‐based bioprinting uses different photoinitiators and UV light during the bioprinting process for crosslinking the hydrogel (Ng et al., 2020). Although this technique allows for the creation of high‐resolution 3D constructs, the UV light used for crosslinking can damage the cells with a consequent reduction in the ability of cells to proliferate and differentiate.

Given such premises, also in the case of BioP for muscle regeneration, the selection of appropriate biomaterials and the resulting bioink is vital to obtain desired biological outcomes. Among the various solutions proposed by the literature and thanks to their features, hydrogels combined with MP cells (C2C12), are commonly used as bioink for skeletal muscle regeneration (Langridge et al., 2021; Malda et al., 2013). In fact, hydrogels are known to be material with high biocompatibility and biodegradability. In addition, their mechanical properties could be modulated by the amount of chemical, temperature, or photo‐crosslinking, to modify the elastic modulus to be as much similar as skeletal muscle tissue (Fischer et al., 2020). Hydrogel‐based bioinks interact with cells in vitro and in vivo, so their viscosity may be optimized to maintain cell integrity and viability during the printing process. For this purpose, it is possible to use natural (chitosan, alginate, collagen, fibrin, etc.) and synthetic (Pluronic F127, poly(ethylene glycol), etc.) polymers that provides cells with an ECM‐like environment (Duarte Campos et al., 2013; Pati et al., 2014; Skardal & Atala, 2015).

Mozetic et al. (2017) engineered the alignment C2C12 by printing Pluronic/alginate composite hydrogel to fabricate highly organized structures that could be used for the assembly of an entire muscle by pneumatic extrusion‐based technique. On the other hand, Kim and colleagues combined dECM methacrylate (dECM‐MA) derived from porcine skeletal muscles with fibrillated polyvinyl alcohol to fabricate a uniaxial oriented dECM‐MA patterned structure of C2C12 by pneumatic extrusion‐based technique (Kim et al., 2020).

Consequently, such a variety bioinks and related 3D manufacturing protocols proposed in the literature calls for a systematic investigation and comparison of different biomaterial ink for 3D BioP in muscle regenerative medicine. Given this motivation, the present study proposes to investigate the viability, proliferation, and differentiation of 3D bioprinted murine myoblast (C2C12) laden into three different types of commercial hydrogels crosslinked with different approaches.

## 2 MATERIALS AND METHODS

Murine myoblasts were mixed with three commercial hydrogels (CELLINK^®^ GelMA A, CELLINK^®^ GelXA FIBRIN, CELLINK^®^ FIBRIN) and extruded by pneumatic extrusion‐based bioprinter (INKREDIBLE+^®^). In the resulting constructs, C2C12 proliferation and differentiation were analyzed at different time points (24 h, 7, 14, 21, and 28 days) using morphological tests (Live/Dead staining and immunofluorescence [IF]). Molecular biology tests were also performed to quantify the gene expression of specific myogenic markers involved in muscle fiber maturation.

### 2.1 Cell culture

C2C12 myoblasts (purchased from ATCC, CRL‐1772 ™) were cultured in DMEM supplemented with 10% fetal bovine serum, 1% penicillin/streptomycin (Sigma), 1% glutamine and 2% sodium pyruvate at 37°C under a 5% CO_2_ atmosphere. When 80% cell density was reached, cells were used for the experiments. C2C12 concentration in the bionks was approximately 25 × 10^6^ C2C12 cells/mL. Cell counting was performed using a Burker's chamber and an Eclipse TE200 microscope (Nikon).

### 2.2 Hydrogels and crosslinkers

The experiments were performed using commercially available Gelatin‐based hydrogel and alginate (CELLINK^®^ GelMA A), Xantan gum and Fibrinogen hydrogel (CELLINK^®^ GelXA FIBRIN) and nanofibrillated cellulose (NFC)/alginate‐fibrinogen‐based hydrogel (CELLINK^®^ FIBRIN).

*Gelatin‐based and alginate hydrogel* (CELLINK^®^ GelMA A). The chemical composition of this hydrogel is a blend of CELLINK® GelMA and alginate, offering a higher printability compared to pure CELLINK^®^ GelMA hydrogels. This is due to the provided softening of the alginate and to essential properties of native ECM that allow cells to proliferate and spread. CELLINK^®^ GelMA A 3D constructs were crosslinked by photopolymerization, or through the addition of the ionic crosslinking solution (50 mM CaCl2).

*Xanthan gum and Fibrinogen hydrogel* (CELLINK^®^ GelXA FIBRIN). This hydrogel incorporates GelMA base, xanthan gum and alginate to enhance printability and stability of the 3D constructs, while fibrin improves muscle cell proliferation and differentiation. A combination of photoinitiator‐assisted and ionic crosslinking was applied.

*Nanofibrillated cellulose/alginate‐fibrinogen‐based hydrogel* (CELLINK^®^ FIBRIN). This hydrogel contains NFC, alginate, fibrinogen and in situ fibrin to provide a physiologically relevant environment for in vitro muscle tissue generation. In addition, it includes an enhanced crosslinking solution composed by thrombin and ionic binding agent (CaCl_2_) to develop a compound network with suitable printability and stability. The 3D fibrin‐constructs were crosslinked with 50 mM calcium chloride in two rounds of experiments, while in other two rounds were crosslinked with a blend solution of thrombin and calcium chloride.

In Table 1 are summarized the hydrogels and relative crosslinkers used for each round of 3D printing experiments. In addition, rheological tests were carried out directly by CELLINK (CELLINK AB) for each hydrogel (Figure S1).

**TABLE 1:** Summary of hydrogels and crosslinkers used in the experiments

Hydrogels	CaCl_2_	Thrombin	UV
CELLINK^®^ GelMA A	✔	✔	‐‐‐
CELLINK^®^ GelXA FIBRIN	✔	‐‐‐	✔
CELLINK^®^ FIBRIN	✔	✔	‐‐‐

### 2.3 3D bioprinter

The CELLINK INKREDIBLE+ (CELLINK AB, Gothenburg, Sweden) is a pneumatic extrusion‐based 3D bioprinter with dual heated print heads which can be heated up to a maximum of 130°C and UV LED curing system (365 and 405 nm). The INKREDIBLE+ is equipped with a patented Clean Chamber Technology and high efficiency particulate air (HEPA) filtered positive air pressure inside the printing chamber. The sterility of the printing chamber was guaranteed by activating 365 nm UV light, positive pressurized airflow and the HEPA H13 filter. The BioP process works through the layer‐by‐layer extrusion of the biomaterial, with a viscosity range between 0.001 and 250 Pa/s.

### 2.4 Bioprinting process

Before starting the printing process, the bioprinter was placed under a sterile hood and UV light was turned on for 1 h to sterilize all the materials and surfaces. Hydrogel was mixed with C2C12 cells (10:1 ratio). The Cartridge was filled with bioink, then nozzle connected (inner diameter 0.25 mm) and finally placed into the printhead. The axes were homed, the *z*‐axis was calibrated, and the pressure and printing speed was set according to standard guidelines (10–15 kPa and 1000 mm/min respectively for all bioinks tested). The 3D constructs were bioprinted on a *Petri* dish, then the crosslinking process was performed as follows. For chemical crosslinking, CaCl_2_ droplets were applied to cover the whole 3D structure and immediately after, the samples were incubated for 5 min at room temperature (RT). The crosslinking solution was subsequently removed from the constructs and DMEM culture complete medium was added. Dishes were then incubated at 37°C and 5% CO_2_. Only the chemical crosslinking process was repeated weekly before medium refreshment to keep the three‐dimensional structure unchanged and avoiding degradation. For UV crosslinking, 3D constructs were exposed once to UV light at 365 nm for approximately 3/5 s.

### 2.5 3D structure

**FIGURE 1:**
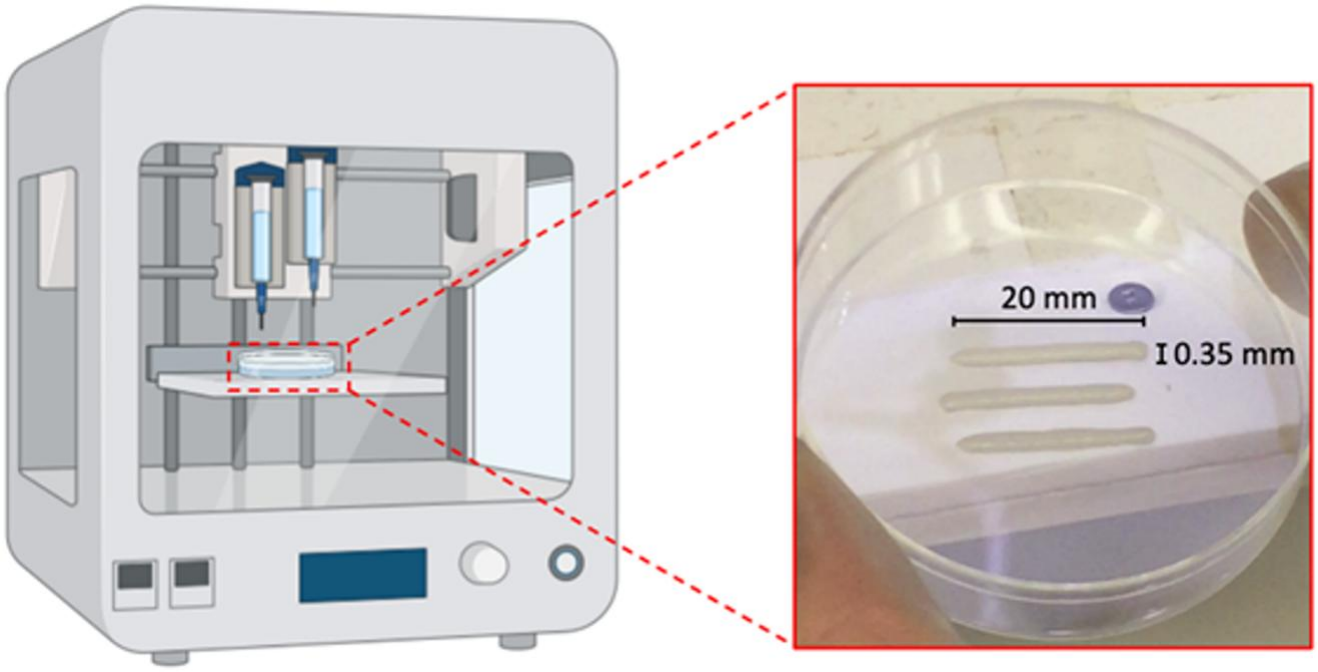
Schematic representation of the bioprinting process with focus on the 3D geometry lines formed by one layer

To mimic morpho‐physiology muscle fiber structure, 3D geometry lines formed by one layer were bioprinted. Line length was set at 20 mm, while the line thickness is given by the combination of pressure and printing speed. In this case, it is equal to 0.35 mm (Figure 1). Given the simplicity of the structure considered, we directly implemented the G‐code of the 3D virtual model.

### 2.6 Cell culture of 3D constructs

3D bioprinted constructs were cultured up to 28 days in DMEM complete medium at 37°C and 5% CO_2_. The culture medium was refreshed every 3 days. 3D constructs were crosslinked every 3 days for 5 min. Following 4 days of BioP, the differentiation process of C2C12‐laden bioink was induced by using a differentiation medium (DM) composed by DMEM supplemented with 2% fetal bovine serum.

### 2.7 Live/dead staining

To evaluate cell viability, we used the Live/Dead staining (Invitrogen); 500 μL of a solution consisting of 1.5 ml of Phosphate Buffered Saline (PBS), 3 μL of EthD‐1 and 1.5 μL of calcein, was added to 3D constructs. Samples were incubated for 45 min in the dark, then the solution was removed, and cell nuclei were counterstained with 500 μL 4′,6‐diamidino‐2‐phenylindole (DAPI) for 10 min according to the protocol. Fluorescent image acquisition was carried out by semi‐confocal microscope (ViCo confocal, Nikon).

Viability and differentiation tests were performed as well as morphological and gene expression analysis at six different time points (1, 4, 7, 14, 21, and 28 days in culture).

### 2.8 Total RNA extraction and quantitative real‐time PCR

Expression levels of myogenic genes were analyzed on 3D bioprinted constructs by Quantitative real‐time PCR (RT‐qPCR).

Total RNA derived from each sample was extracted and isolated at different time points using 300 μL of lysis buffer (TRIzol Reagent). Total RNA extraction was performed by using Direct‐zol RNA Miniprep's reagents following the manufacturer protocol (Zymo Research). Total RNA was then quantified by NanoDropTM (Thermo‐Fisher Scientific). cDNAs obtained from 350 ng of RNA were reverse transcribed using iScript™ cDNA Synthesis Kit (Biorad) and quantitative PCR analysis was performed using oligonucleotide primers displayed in Table 2. The reaction was carried out by using MiniOpticon Real‐Time PCR System (BioRad Laboratories) and data analysis was performed by CFX Manager Software. Gene expression was analyzed in triplicate and normalized to glyceraldehyde 3‐phosphate dehydrogenase (PGK) gene expression. In order to elucidate the differentiation process of C2C12‐laden bioinks, the gene expression profile of relevant myogenic differentiation markers was evaluated (MyoD, Muscle creatine kinase [MCK]).

**TABLE 2:** Summary of primers used for quantitative PCR analysis

Gene names	Primer sequences
PGK (115 bp)	Fw 5′ CAA AAT GTC GTC TTC CAA CAA G 3′
	Rw 5′ AAC GTT GAA GTC CAC CCT CAT 3′
MyoD (115 bp)	Fw 5′ TGCACTTCCACCAACCCCAACCAGC 3′
	Rw 5′ CCTGGACTCGCGCACCGCCTCACT 3′
MCK (103 bp)	Fw 5′ CCTGTTTGATCCCATCATCC 3′
	Rw 5′ AGCACATAGTTGGGGTCCAG 3′

### 2.9 Immunofluorescence assay

Immunofluorescence assay on in vitro 3D constructs was performed to evaluate morphologically the differentiation of C2C12 cells laden into different bioinks at 7, 14, 21 and 28 days of culture in DM. 3D constructs were blocked with PAT (PBS containing 1% [w/v] bovine serum albumin (BSA) and 0.02% [v/v] Tween 20) solution for 1 h at RT. Subsequently, samples were incubated with MF20 primary antibody (Myosin Heavy Chain Antibody, diluted in PBS‐Tween 0.1% and in BSA 1% 1:20) for 1 h at RT. After several washes with buffer solution, sections were incubated with a secondary antibody diluted in 0.1% PBS‐Tween and 1% BSA (1:1000), and in diluted Phalloidine (1:40). Samples were counterstained with DAPI to detect nuclei, washed three times with a washing buffer, and ultimately mounted. Finally, sections were observed with a semi‐confocal microscope (ViCo confocal, Nikon), supported by the ImageJ PRO 6.2 software.

## 3 RESULTS

### 3.1 Live/dead staining

**FIGURE 2:**
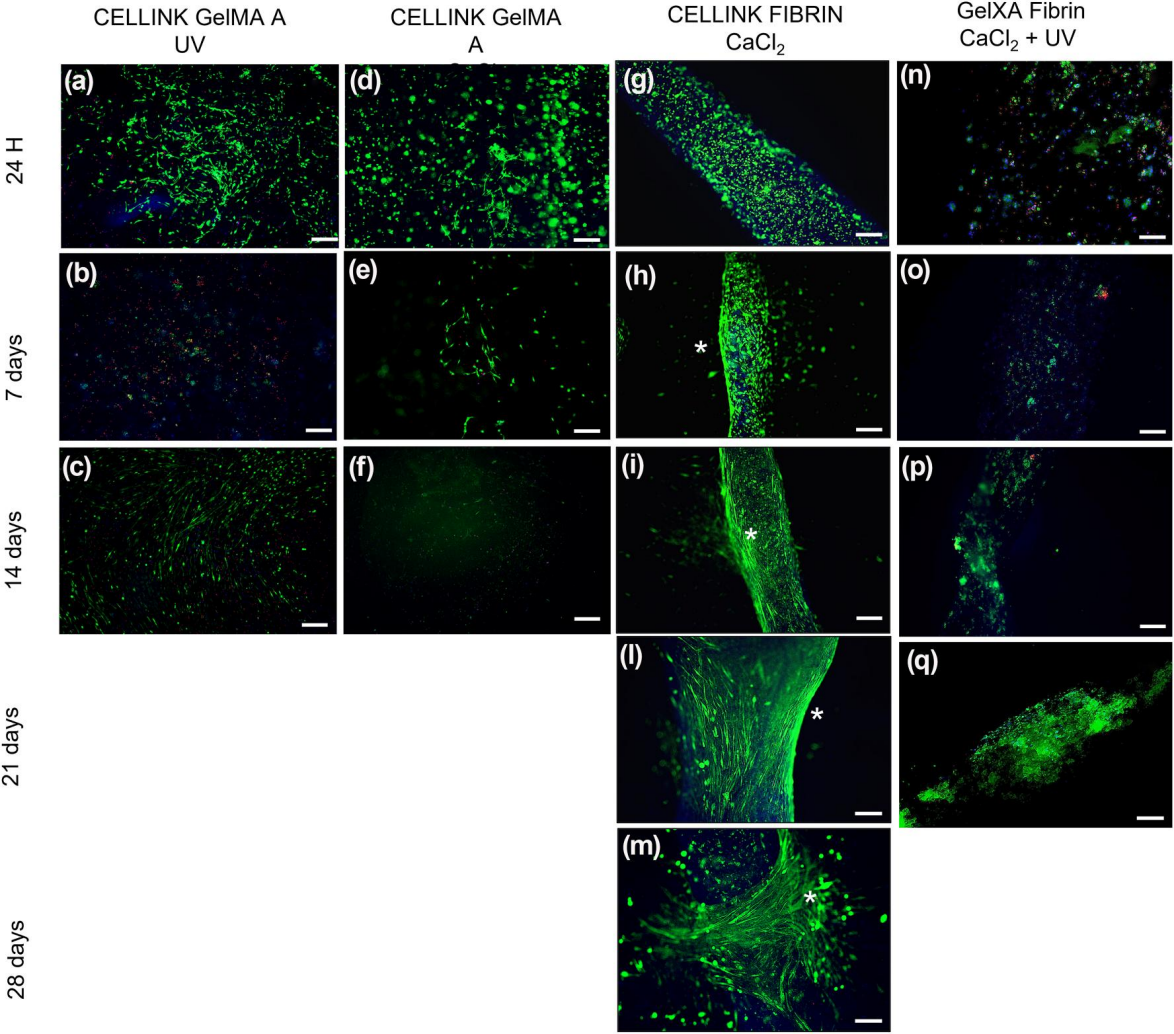
Live (green)/Dead (red) and 4′,6‐diamidino‐2‐phenylindole (blue) images of different bioinks at specific time points in proliferative conditions. (a–c) CELLINK^®^ GelMA A‐UV 3D constructs; (d–f) CELLINK^®^ GelMA A CaCl2 3D constructs (g–m) CELLINK^®^ FIBRIN 3D constructs; (n–q) CELLINK^®^ GelXA FIBRIN 3D constructs. Due to mold contamination on construct borders, the experiments for CELLINK^®^ GelXA FIBRIN and CELLINK^®^ GelMA A have been prematurely interrupted on days 21 and 14 respectively. Scale bar 50 μm. Cell elongation is highlighted by asterisks (*)

**FIGURE 3:**
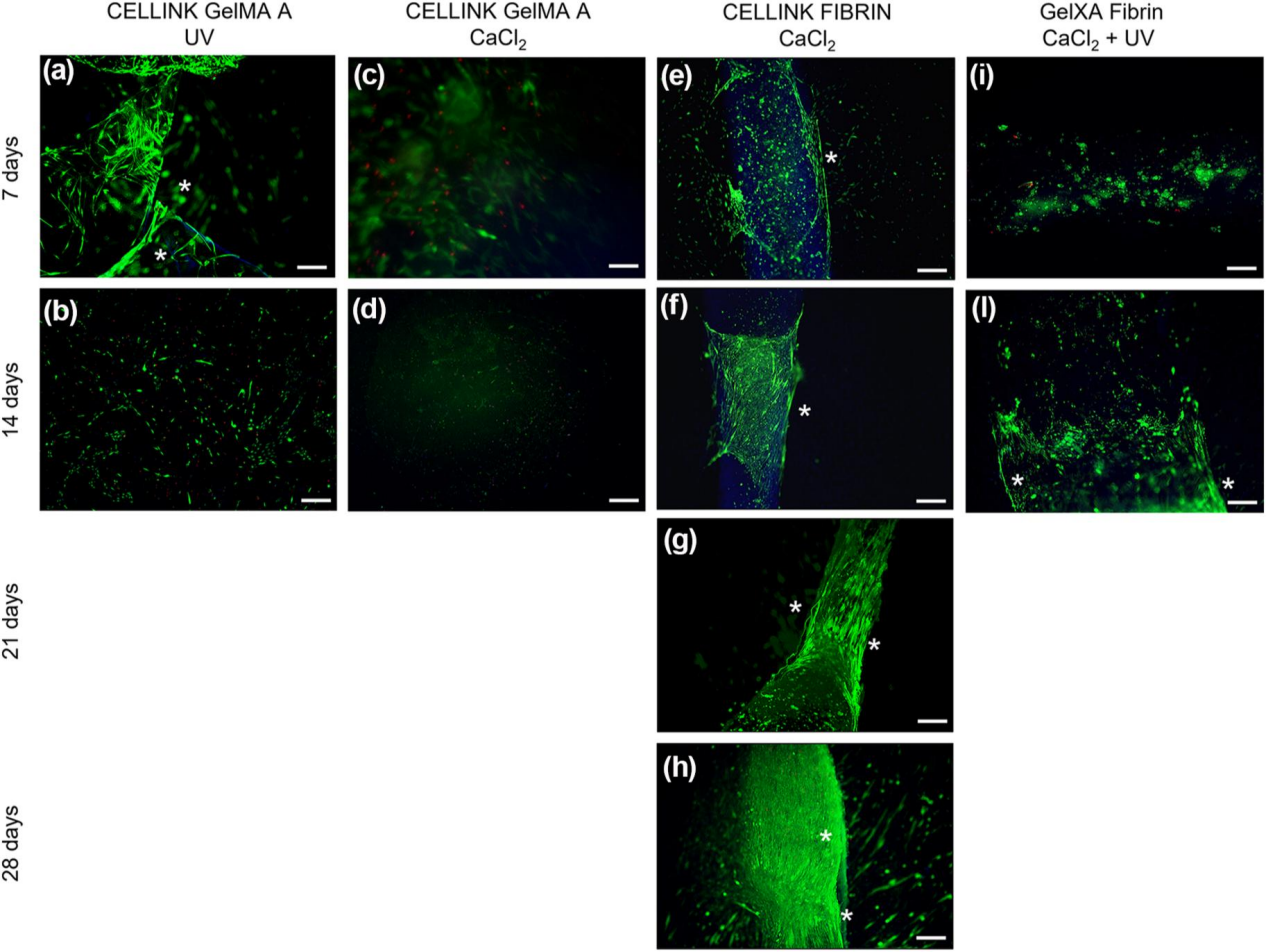
Live (green)/Dead (red) and 4′,6‐diamidino‐2‐phenylindole (blue) images of different bioinks during differentiation. (a,b) CELLINK^®^ GelMA A‐UV 3D constructs; (c,d) CELLINK^®^ GelMA A CaCl2 3D constructs (e–h) CELLINK^®^ FIBRIN 3D constructs; (i–l) CELLINK^®^ GelXA FIBRIN 3D constructs; Scale bars 50 μm. Cell elongation is highlighted by asterisks (*)

Live/Dead staining was performed at different time points during both cell proliferation (Figure 2) and differentiation (Figure 3).

C2C12 cells encapsulated in CELLINK^®^ GelMA A hydrogel crosslinked with CaCl_2_ and UV light showed a poor myotubes formation, not statistically significant compared to CELLINK^®^ FIBRIN hydrogel (Figure 2a–f). Particularly, at 7 and 14 days in culture, we observed that CaCl_2_ and UV light exposure enhanced myoblast elongation, but not in a homogeneous trend (Figure 2b–f). We observed that cell viability was approximately 90% during all time points.

At 24 h, 94% cell viability was detected in CELLINK^®^ FIBRIN hydrogel (Figure 2g).

Seven days after the BioP, C2C12 rapidly spread within the hydrogel matrices, remaining mainly round shaped in the center of the construct (Figure 2h). Nevertheless, especially in CELLINK^®^ FIBRIN 3D constructs, an initial C2C12 differentiation began at the borders of the 3D constructs, where small myotube formation appeared. Whereas, in the central part of the 3D structure C2C12 cells were not merged forming myotubes. This is probably due to a non‐homogeneous diffusion of the crosslinking solution or to lower oxygen and nutrient levels within the 3D constructs (Figure 2i).

Finally at 21 and 28 days, C2C12 cells merged forming myotubes even in the most central part of the 3D structure, and the alignment was promoted by the linear shape of the printed construct (Figures 2l,m).

Regarding C2C12 cells laden in CELLINK^®^ GelXA hydrogel, 94% viability was observed at all the time points analyzed (Figure 2n–o). Nevertheless, at 7 and 14 days in culture, cells kept a round shape and slowly start to elongate only at day 21 especially at the borders of the constructs (Figure 2o–q).

Live/Dead staining in proliferative conditions was also performed on CELLINK^®^ FIBRIN 3D constructs, crosslinked with CaCl_2_ and Thrombin. We observed no advantages on cell viability, adhesion, spreading, and differentiation (data not shown).

In Figure 3 we reported Live/Dead staining of 3D constructs in DM.

In all the time points and for all bioinks, cells demonstrated high viability (>94%). The differentiation rate of the C2C12 cells decreased significantly when printed in CELLINK^®^ GelMA A (Figure 3a–d) and CELLINK^®^ GelXA FIBRIN hydrogels (Figure 3i–l), in fact they did not show any improvement in the myogenic differentiation process. Otherwise, CELLINK^®^ FIBRIN hydrogel appeared to increase significantly the myotube formation, and the myoblast alignment is principally located at the borders, as well as in the proliferative condition (Figure 3E–h). Particularly, at day 28 C2C12 cells differentiated forming myotubes that covered the whole 3D linear structure (Figure 3h).

Thus, Live/Dead results indicated that in proliferative and differentiative conditions the CELLINK^®^ FIBRIN hydrogel effectively induced myoblast alignment particularly at the border of the structure in comparison to the other hydrogel tested. The lower nutrient/oxygen diffusion or a possible non homogenous spreading of crosslinking agent to the center of the construct could be responsible of a non‐sufficient myotube formation.

### 3.2 Immunofluorescence analysis

**FIGURE 4:**
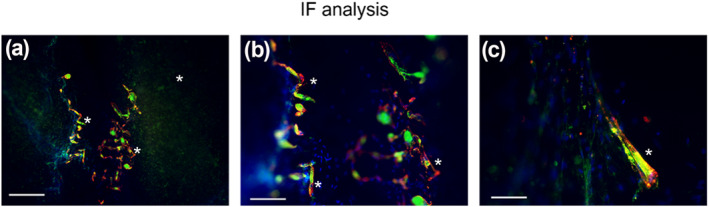
IF assay of CELLINK^®^ FIBRIN hydrogel by actin (green) and M‐cadherin (red) after 28 days of skeletal muscle differentiation. Nuclear staining by 4′,6‐diamidino‐2‐phenylindole (blue). Scale bars 100 μm (a), 50 μm (b), 10 μm (c). Myotubes are highlighted by asterisks (*)

Differentiation of C2C12 cells was also analyzed by IF analysis after 28 days in culture, as shown in Figures 4a–c.

Immunofluorescence staining confirmed myoblast fusion and myotube formation at the border of the CELLINK^®^ FIBRIN 3D construct. In this regard, myoblast alignment and fusion were identified with the colocalization of MF‐20 antibody and Phalloidin immunoreactivity molecule (Figure 4a); differentiation rate and myotube formation were detected by actin‐positive signals (Figures 4b,c).

The Figures 4a–c show how the CELLINK^®^ FIBRIN hydrogel induced myoblast alignment especially at the border of the 3D construct (color merge).

### 3.3 Gene expression analysis of cell‐laden structures by quantitative real‐time PCR

**FIGURE 5:**
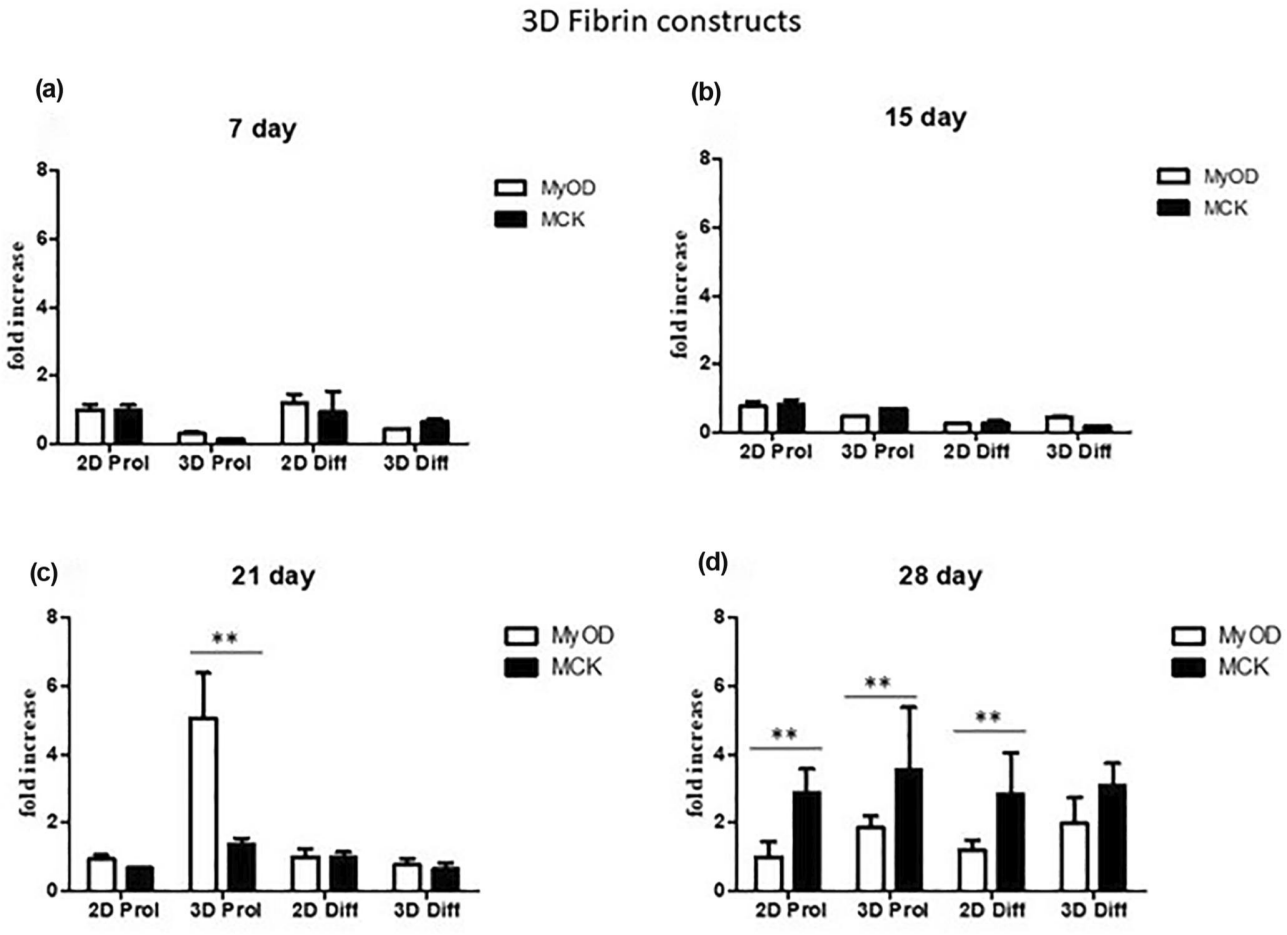
Gene expression analysis of C2C12 laden with CELLINK^®^ FIBRIN hydrogel at 7, 14, 21 and 28 days. (a) qRT‐PCR at 7 days. (b) qRT‐PCR at 14 days. (c) qRT‐PCR at 21 days. (d) qRT‐PCR at 28 days. Results are normalized to the housekeeping gene (3‐phosphate dehydrogenase [PGK]). Statistically significant values are indicated as *0.05 < *P* < 0.01 and **P < 0.01. Analysis of variance test was performed to evaluate data significance

**FIGURE 6:**
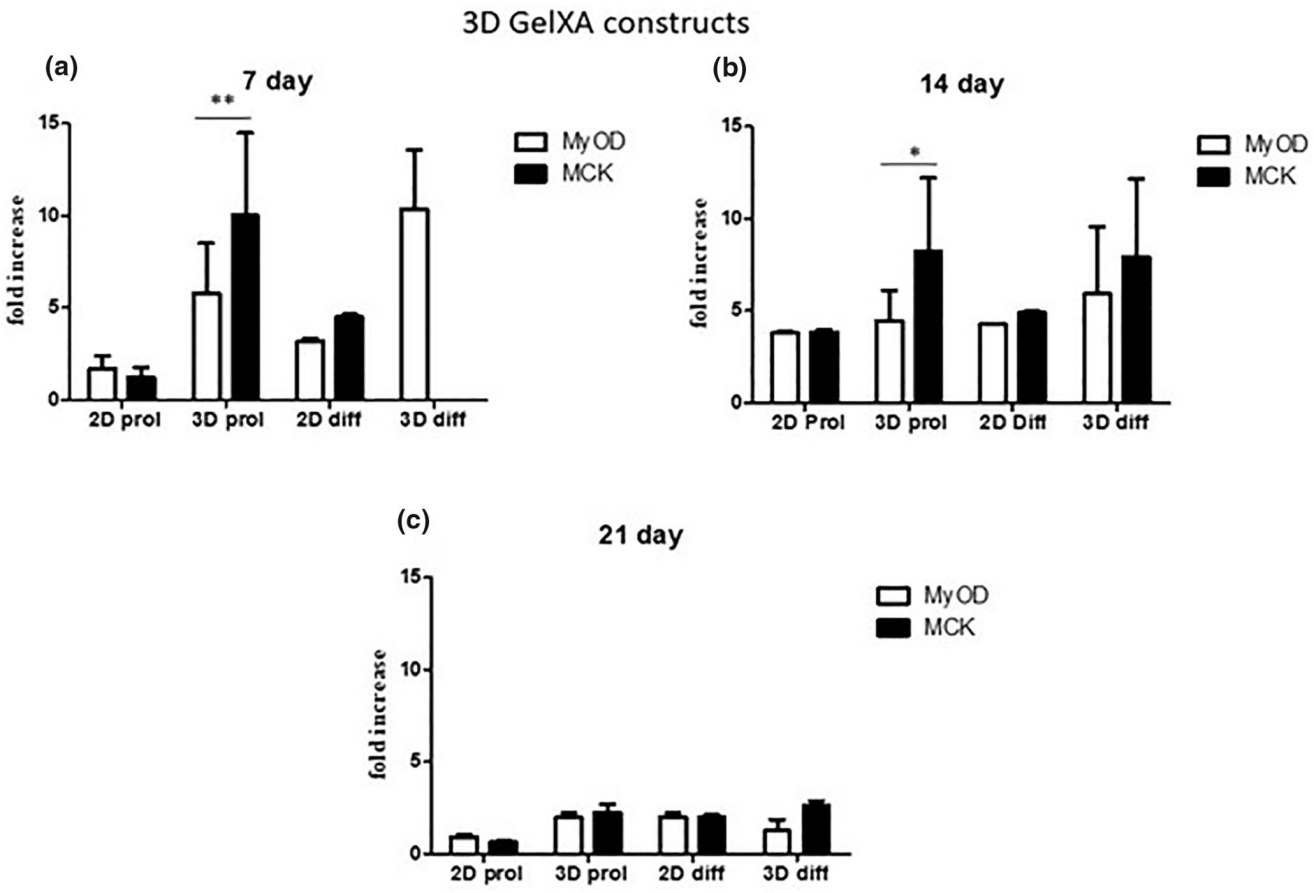
Gene expression analysis of C2C12 laden with CELLINK^®^ GelXA FIBRIN hydrogel at 7, 14 and 21 days. (a) qRT‐PCR at 7 days. (b) qRT‐PCR at 14 days. (c) qRT‐PCR at 21 days. Results are normalized to the housekeeping gene (3‐phosphate dehydrogenase [PGK]). Statistically significant values are indicated as *0.05 < *P* < 0.01 and **P < 0.01. Analysis of variance test was performed to evaluate data significance

Gene expression analyses were performed to evaluate and validate the observed differentiation rate of C2C12 cells laden into CELLINK^®^ FIBRIN hydrogel at 7, 14, 21, and 28 days and into CELLINK^®^ GelXA FIBRIN hydrogel at 7, 14, and 21 days in culture in proliferative and differentiative conditions (Figure 5 and Figure 6).

The expression levels of myogenic genes such as MyoD and MCK in the 3D structures were detected by RT‐qPCR normalized by the PGK gene.

Regarding the CELLINK^®^ FIBRIN hydrogel, after 7 days of culture in proliferative conditions, MyoD and MCK were expressed 1.2‐fold higher than in 3D cultures (Figure 5a).

Similarly, after 7 days in DM, the expression of both genes was 1.8‐fold higher in 2D than in 3D (Figure 5a, *p* > 0.05). Thus, at the initial phase of culture, the 3D seems not to enhance the differentiation process of the myogenic cells.

After 15 days, gene expression of MyoD and MCK genes was comparable to day 7 and no statistical significance of 3D samples versus controls was detected (Figure 5b). At 21 and 28 days of culture, as indicated also by Live/Dead staining, the expression levels of myogenic genes were significantly higher compared to controls, especially for MCK gene in all 3D structures (Figure 5c–d, *p* < 0.01), during both proliferation and differentiation. Probably, this enhancement of the myogenic gene signature is due to the biochemical and topographical cues of CELLINK^®^ FIBRIN hydrogel clearly inducing highly efficient myoblast differentiation compared to 2D.

On the contrary, gene expression of myogenic genes in C2C12 cells laden with CELLINK^®^ GelXA FIBRIN hydrogel seems to be upregulated at 7 and 14 days of culture compared to 2D samples (Figure 6a, *p* < 0.01 and Figure 6b). At day 21, no statistical differences were observed between 2D and 3D samples (Figure 6c).

Gene expression was also evaluated for the other hydrogel (CELLINK^®^ GelMA A), but no statistical differences were highlighted among the samples (data not shown).

In conclusion, CELLINK^®^ FIBRIN hydrogel, as indicated also by Live/Dead staining, improves myogenic gene signature, and proves to be the best bioink to promote myoblast alignment along the printed filament.

## 4 DISCUSSION

In this study, we demonstrated the impact of different types of hydrogels on the viability, proliferation, and differentiation of murine myoblasts encapsulated in 3D constructs and manufactured by pneumatic extrusion based BioP.

Skeletal muscle tissue engineering characterizes a revolutionary branch of regenerative medicine which aims to recreate in vitro muscles to be studied ex vivo and ultimately for the substitution of diseased or damaged muscle tissue.

Up to the present time, many different strategies have been proposed even if not fully suitable for a potential therapeutic application (Fuoco et al., 2016; Levenberg et al., 2005; Shadrin et al., 2016; Sicari et al., 2014). One of the main issues encountered was the identification of the best hydrogel to achieve sarcomerogenesis and the parallel‐oriented myofiber organization resembling the correct skeletal muscle structure. Therefore, to improve skeletal muscle tissue engineering, innovative techniques are required to produce engineered constructs with precise 3D structures. To date, pioneering technologies are revolutionizing many different manufacturing fields, including tissue engineering (Costantini et al., 2018). Especially 3D BioP techniques showed a prodigious potential for the rapid and cost‐effective fabrication of cellularized structures, to build human‐sized myo‐constructs (Agosti et al., 2020; Mozetic et al., 2017; Ott et al., 2008). Although in this study we focused on extrusion‐based bioprinting there are other works hat use different technique such as inkjet and vat polymerization. For example, inkjet‐based bioprinting was used to fabricate biocompatible substrates used for fabricating an electrostimulation device to guide cell alignment and enhance myotubes differentiation (Fortunato et al., 2018).

In this study, we tested multiple commercially available hydrogels characterized by specific composition and rheological capabilities to understand which is the best biomaterial that promotes the formation of a functionalized myo‐construct. We did not perform a thorough rheological characterization of the different bioinks used to understand the shear stress experienced by the cells during the printing process (Lucas et al., 2020; Mondal et al., 2019; Ning et al., 2018; Serna et al., 2019), but this aspect could be explored by future development of the present study.

We demonstrated the successful 3D BioP of CELLINK^®^ FIBRIN and CELLINK^®^ GelMA A hydrogels also implemented with photocurable biopolymers. All biomaterials tested in this study showed the capacity to facilitate skeletal muscle cell survival and differentiation with different degrees of efficiency. CELLINK^®^ FIBRIN hydrogel exhibited the best printability performances with highest handiness for the operators. Even though, CELLINK^®^ GelMA A hydrogel shows an excellent printability at RT; in our experiment it shows to be less efficient than CELLINK^®^ FIBRIN and CELLINK^®^ GelXA FIBRIN hydrogels that could be both used to potentially stimulate the myoblast activation and differentiation when used as a 3D matrix. This could be related to the specific formulation and structure of each biomaterial. The internal structure of the hydrogels is crucial to metabolite transport inside the 3D constructs. Nutrient, oxygen and protein spreading, as well as cell migration and differentiation are supported by diffusion within any matrix with embedded cells (Visser et al., 2013).These findings denote a remarkable improvement, as it has been shown that fibrinogen‐related biomaterials stimulate cell adhesion, spreading, and differentiation of multiple cell sources including myogenic progenitor cells, especially due to their biodegradable and non‐immunogenic features (Almany & Seliktar, 2005; Centola et al., 2013; Fuoco et al., 2012, 2014, 2015). These characteristics, joint with the hydrogels composed of Fibrinogen/Gelatin allowed the creation of myo‐constructs containing myogenic progenitors (C2C12) in precisely defined constructs promoting myotube formation and alignment (Figures 2 and 3). Even if recent studies investigated 3D printing techniques for skeletal muscle tissue engineering (Karande et al., 2004; Mironov et al., 2008), the results achieved were still poor, highlighting an unsatisfactory structural organization both in vitro and in vivo. Conversely, in this paper we showed a significant morphological organization of the myotubes, resembling mature sarcomerogenesis (Figure 4). Finally, while the use of any of these hydrogels requires further optimization to maximize their functional and myogenic properties, the obtained results provide a knowledge advance in the field and a promising tool for skeletal muscle tissue engineering.

## 5 CONCLUSION

We performed a comparative study of hydrogel behavior testing their myogenic properties over a long‐time course (28 days) to analyze how the biomaterial matrix could improve muscle precursor cell (C2C12) viability and differentiation. The linear 3D printed structures were tested in vitro to assess their ability to stimulate myogenesis. Our results clearly showed that CELLINK^®^ FIBRIN and slightly less CELLINK^®^ GelXA FIBRIN hydrogels demonstrated the best potential to support the in vitro long‐term differentiation of skeletal muscle cells in 3D constructs. After 21–28 days in culture, myogenic cells were able to fuse together forming structurally aligned myotubes, with high expression levels of specific skeletal muscle markers such as Myogenic Differentiation 1 and MCK genes.

Due to all these findings, the results reported herein denote a significant enhancement to improve skeletal muscle tissue engineering.

## CONFLICT OF INTEREST

The authors declare that the research was conducted in the absence of any commercial or financial relationships that could be construed as a potential conflict of interest.

## AUTHOR CONTRIBUTIONS

Flavio L. Ronzoni, Flaminia Aliberti, Gabriele Ceccarelli, Franca Scocozza, Laura Benedetti and Michele Conti designed and performed experiments, and analyzed data. Maurilio Sampaolesi, Ferdinando Auricchio, Michele Conti and Gabriella Cusella provided funding. Flavio L. Ronzoni, Flaminia Aliberti, Gabriele Ceccarelli, and Michele Conti drafted the manuscript. All authors have read and agreed to the published version of the manuscript.

## Supporting information

Supplementary Material 1

## Data Availability

The data that support the findings of this study are available from the corresponding author upon reasonable request.
