# Supplementary figures and images for: Myoblast 3D bioprinting to burst in vitro skeletal muscle differentiation
Source: J Tissue Eng Regen Med. 2022 Mar 4;16(5):484–95. doi: 10.1002/term.3293 (PMC9311434; doi:10.1002/term.3293)

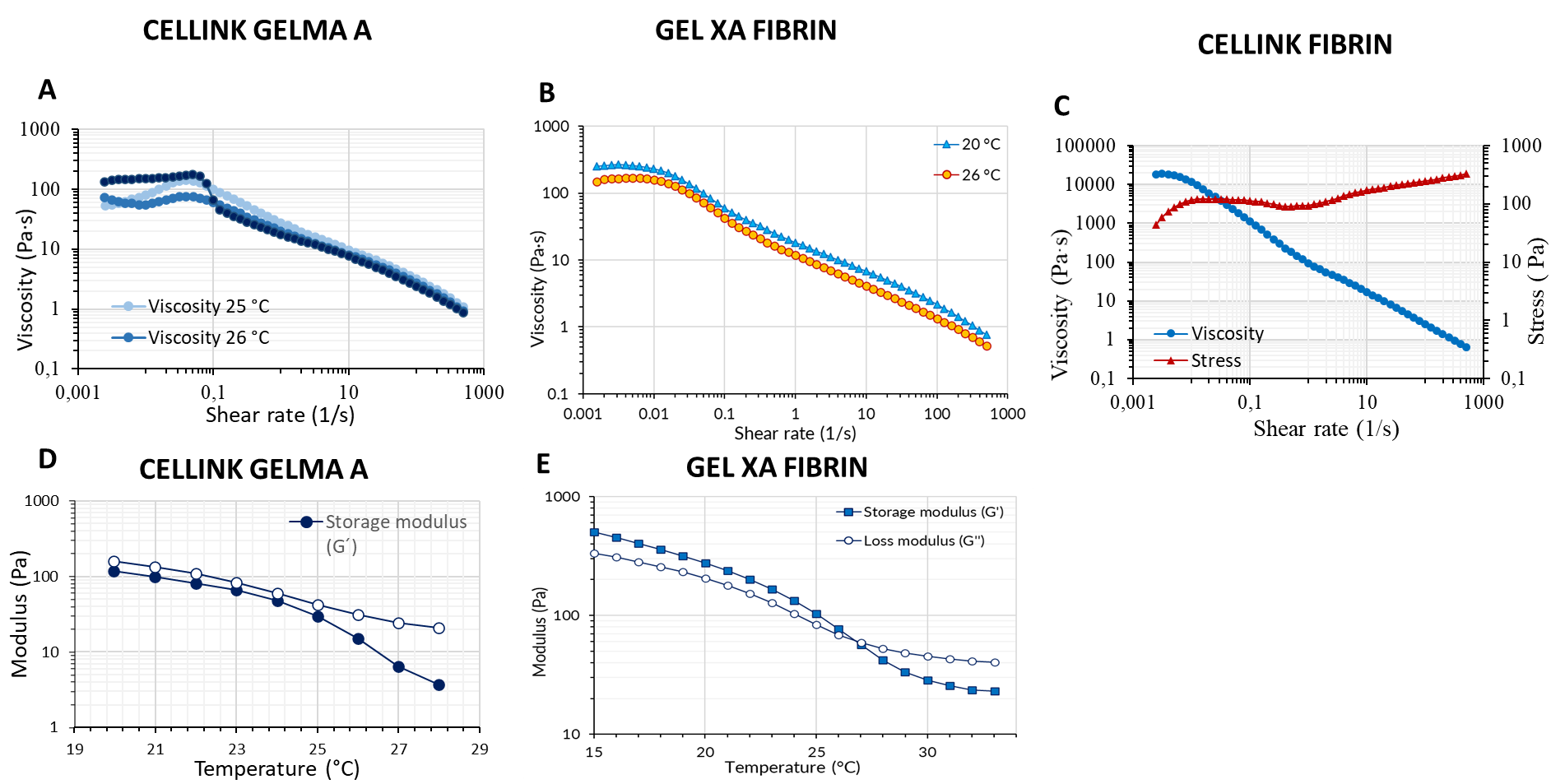

Supplement: Supplementary file 1 — Supplementary Material 1 [file TERM-16-484-s001.tif]
